# The *Meta*-Substituted Isomer of TMPyP Enables More Effective Photodynamic Bacterial Inactivation than *Para*-TMPyP In Vitro

**DOI:** 10.3390/microorganisms10050858

**Published:** 2022-04-21

**Authors:** Sebastian Schulz, Svitlana Ziganshyna, Norman Lippmann, Sarah Glass, Volker Eulenburg, Natalia Habermann, Ulrich T. Schwarz, Alexander Voigt, Claudia Heilmann, Tobias Rüffer, Robert Werdehausen

**Affiliations:** 1 Department of Anesthesiology and Intensive Care, Medical Faculty, University of Leipzig, 04103 Leipzig, Germany; sebastian.schulz@medizin.uni-leipzig.de (S.S.); svitlana.ziganshyna@medizin.uni-leipzig.de (S.Z.); volker.eulenburg@medizin.uni-leipzig.de (V.E.); 2 Institute of Medical Microbiology and Virology, Medical Faculty, University of Leipzig, 04103 Leipzig, Germany; norman.lippmann@medizin.uni-leipzig.de; 3 Leibniz Institute of Surface Engineering (IOM), 04318 Leipzig, Germany; sarah.glass@hereon.de; 4 Institute of Physics, Chemnitz University of Technology, 09111 Chemnitz, Germany; natalia.habermann@s2014.tu-chemnitz.de (N.H.); ulrich.schwarz@physik.tu-chemnitz.de (U.T.S.); 5 Institute of Chemistry, Faculty of Natural Sciences, Chemnitz University of Technology, 09111 Chemnitz, Germany; alexander.voigt@s2012.tu-chemnitz.de (A.V.); claudia.heilmann@mvtat.tu-freiberg.de (C.H.)

**Keywords:** antimicrobial resistance, multi-drug resistance, bacterial infections, photodynamic therapy, photodynamic inactivation, photosensitizer, cationic porphyrins, TMPyP, crystallographic characterization

## Abstract

Porphyrinoid-based photodynamic inactivation (PDI) provides a promising approach to treating multidrug-resistant infections. However, available agents for PDI still have optimization potential with regard to effectiveness, toxicology, chemical stability, and solubility. The currently available photosensitizer TMPyP is provided with a *para* substitution pattern (*para*-TMPyP) of the pyridinium groups and has been demonstrated to be effective for PDI of multidrug-resistant bacteria. To further improve its properties, we synthetized a structural variant of TMPyP with an isomeric substitution pattern in a *meta* configuration (*meta*-TMPyP), confirmed the correct structure by crystallographic analysis and performed a characterization with NMR-, UV/Vis-, and IR spectroscopy, photostability, and singlet oxygen generation assay. *Meta*-TMPyP had a hypochromic shift in absorbance (4 nm) with a 55% higher extinction coefficient and slightly improved photostability (+6.9%) compared to *para*-TMPyP. Despite these superior molecular properties, singlet oxygen generation was increased by only 5.4%. In contrast, PDI, based on *meta*-TMPyP, reduced the density of extended spectrum *β*-lactamase-producing and fluoroquinolone-resistant *Escherichia coli* by several orders of magnitude, whereby a sterilizing effect was observed after 48 min of illumination, while *para*-TMPyP was less effective (*p* < 0.01). These findings demonstrate that structural modification with *meta* substitution increases antibacterial properties of TMPyP in PDI.

## 1. Introduction

Despite available antiseptics and disinfectants, bacterial infections represent a growing major health problem. Traditional antibiotic treatments increasingly fail since antimicrobial resistance (AMR) is a wide-spread phenomenon and a serious global problem [1,2,3,4,5]. It is estimated that the increase in the spread of AMR will lead to a significant increase in mortality and the burden on the global health system in the next few decades [6,7,8]. Already, the use of reserve antibiotics is becoming increasingly necessary in both outpatient and clinical settings because of the high rate of AMR [9,10]. The treatment options for complicated, life-threatening infections caused by multi-resistant pathogens, which are now regularly encountered in intensive care medicine, are severely limited by the decreasing number of remaining effective antibiotics [11]. Furthermore, the so-called last-resort antibiotics often have a considerable adverse effects profile. There has been very little progress in the development of new antibiotic classes or mechanisms of action in recent years [12,13]. Therefore, alternative strategies to control bacteria that are resistant to many antimicrobial drugs are urgently required.

Photodynamic therapy (PDT) is a medical treatment that has hitherto been primarily used to treat cancer but carries great potential for treating critical infections caused by a variety of pathogens including multidrug-resistant species [14,15,16,17,18]. 

PDT utilizes an entirely different mode of action that is unlike usual antimicrobial drugs. For PDT, three components, which are individually non-toxic—a photosensitizer (PS), light, and molecular oxygen (from surrounding air or water)—have to be combined. Together they initiate a photochemical reaction to generate highly reactive and very short-lived oxygen species such as singlet oxygen (^1^O_2_) [14,19,20] as well as superoxide and hydroxyl radicals [19]. Within a tumor cell, this process can lead to apoptosis, while in infected wounds it leads to inhibition of microbial growth [20]. Since porphyrinoid PSs have a low dark toxicity [21] and their cytotoxic mode-of-action is limited to light-irradiated areas with high PS concentration, they become interesting for clinical treatment of bacterial infections. Their bacteriotoxic effect [22,23] has also been demonstrated to be independent of antimicrobial resistance [24,25,26]. Since this mechanism does not rely on specific target pathways and structures, PDT is potentially effective for all classes of microbes, including archaea, bacteria, fungi, protists, and viruses [17].

Several physical and chemical properties determine whether an agent can be used as a PS and its effectiveness in PDT [23,27,28]. Lipophilicity, and charge in particular, has a major influence on the absorption and intracellular distribution of PS [29]. In antimicrobial applications, especially against Gram-negative bacteria, cationic agents have proven to be particularly effective [30], as they are better absorbed than neutral or anionic substances, because electrostatic interactions between the cationic PSs and anionic components of the cell walls and membranes are effective [27]. 

In the context of optimizing PSs for specific applications [24,28], a detailed characterization and comparison in vitro [31,32,33] as well as in in vivo models [34,35,36] for infections with multidrug-resistant pathogens is the subject of current research. The PSs currently used are almost exclusively from the groups of porphyrins, bacteriochlorins, chlorins, and phthalocyanins [14,37]. A particularly pronounced efficacy against Gram-negative bacteria has been described for the cationic PSs such as TMPyP and THTPS [30,33,38,39,40,41]. In contrast, anionic porphyrin derivatives have been reported to be effective for tumor treatment [24].

Porphyrin derivatives such as chlorins and bacteriochlorins show similar pronounced antimicrobial effects but differ in their optical properties in that they are activated by light of different wavelengths and thus their effects are also characterized by different penetration depths into human tissue [42]. For example, PSs such as THPTS, which are activated at wavelengths around 760 nm, have a higher penetration depth in human tissue and thus allow the treatment of deeper tissue layers [43]. In contrast, TMPyP has an absorption maximum in the wavelength range around 420 nm and is therefore particularly suitable for superficial photodynamic treatment, for example of infected wounds. Modified translucent hydrogels can serve as a carrier system, allowing the release of PSs under local illumination [44,45]. This, and the increasing availability of inexpensive lasers and light-emitting diodes (LED) for optical excitation [46,47], may enable applications of PDT both in cancer therapy and against infections with multidrug-resistant bacteria.

The wide spectrum of opportunities for chemical modifications carries great potential for optimization and tailoring PSs for specific applications [48,49]. The photodynamic effect of synthetic porphyrins can be improved by enhancing absorption in the UV–Vis–NIR region, as well as the quantum yield and the efficiency of the energy transfer from the excited triplet state (T1) of these dyes to molecular oxygen with the formation of singlet oxygen [20,29]. This can be achieved by structural modifications of PSs. For example, previous studies on the structure–activity relationship have demonstrated that halogenated tetrapyrrole derivatives exhibit a higher spin-orbital coupling constant than unmodified analogues, thereby favoring intersystem crossing (ISC) [50,51,52]. Moreover, water solubility of porphyrinoids can be improved by introduction of sulfone groups. This does not change their spectroscopic properties, but the additional substituents improve their stability [53,54].

**Figure 1:**
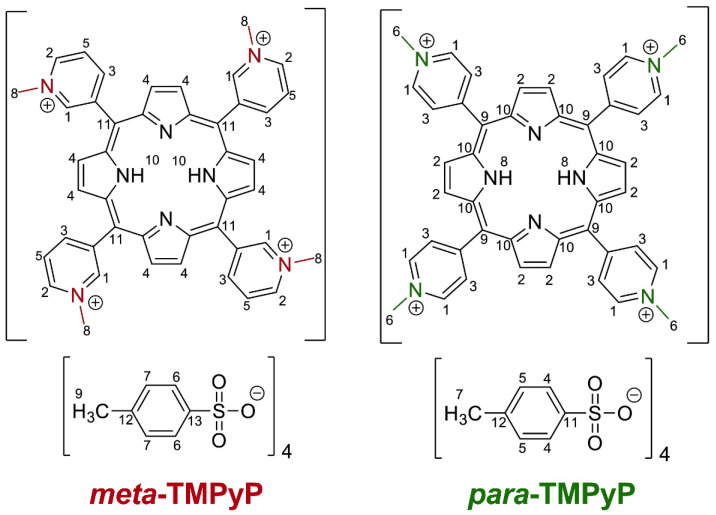
Chemical structures of (3,3′,3″,3‴-(5,10,15,20-tetrayl)tetrakis(1-methylpyridin-1-ium)porphyrin tetra-4-methylbenzenesulfonate (left, ***meta*-TMPyP**) and of (4,4′,4″,4‴-(5,10,15,20-tetrayl)tetrakis(1-methylpyridin-1-ium)porphyrin tetra-4-methyl-benzenesulfonate (right, ***para*-TMPyP**) ^1^H NMR analysis of ***meta*-TMPyP** resulted in the following *δ/*ppm: 10.03 (H-1, d, ^3^*J_H,H_* = 5.4 Hz, 4 H), 9.58 (H-2, d, ^3^*J_H,H_* = 6.2 Hz, 4 H), 9.33 (H-3, d, ^3^*J_H,H_* = 6.9 Hz, 4 H), 9.27 (H-4, s, 8 H), 8.63 (H-5, t, ^3^*J_H,H_* = 7.0 Hz, 4 H), 7.41 (H-6, d, ^3^*J_H,H_* = 8.0 Hz, 8 H), 7.00 (H-7, d, ^3^*J_H,H_* = 7.8 Hz, 8 H), 4.70 (H-8, s, 12 H), 2.19 (H-9, s, 12 H), −3.12 (H-10, s, 2 H). ^13^C{^1^H}-NMR results for ***meta*-TMPyP** were (*δ/*ppm): 145.7 (C-12), 144.5 (C-1), 140.2 (C-3), 138.2 (C-2), 137.7 (C-13), 128.7 (C-5), 128.4 (C-7), 126.8 (C-11), 125.8 (C-6), 113.75 (C-4), 48.7 (C-8), 21.1 (C-9). Infrared spectroscopy of ***meta*-TMPyP** revealed (KBr; *ṽ*/cm^−1^): 3323 w (ν*_N−H_*), 3059 m (ν *_= C−H_*), 3039 m (ν *_= C−H_*), 2955 w (ν *_= C−H_*), 2919 w (ν *_= C−H_*), 1504 w, 1455 w, 1406 w, 1385 w, 1358 vw, 1286 vw, 1214 s, 1193 s ($\nu_{{\mathrm{SO}}_{2}}$), 1121 m, 1033 m (δ*_C−H_*), 1011 m, 980 w, 914 w, 873 w, 816 m, 799 w, 784 w (γ *_= C−H_*), 734 vw, 712 vw, 682 s, 644 w, 570 s.^1^H NMR analysis of ***para*-TMPyP** resulted in the following δ/ppm: 9.47 (H-1, d, ^3^J_H,H_ = 6.6 Hz, 8 H), 9.18 (H-2, s, 8 H), 8.98 (H-3, d, ^3^J_H,H_ = 6.7 Hz, 8 H), 7.45 (H-4, d, ^3^J_H,H_ = 8.0 Hz, 8 H), 7.08 (H-5, d, ^3^J_H,H_ = 7.8 Hz, 8 H), 4.72 (H-6, s, 12 H), 2.25 (H-7, s, 12 H), −3.12 (H-8, s, 2 H). ^13^C{^1^H}-NMR results for ***para*-TMPyP** were (δ/ppm): 156.2 (C-10), 145.8 (C-1), 144.7 (C-11), 137.5 (C-12), 137.1 (C-2), 132.1 (C-3), 128.0 (C-5), 125.5 (C-4), 115.9 (C-9), 47.9 (C-6), 20.7 (C-7). Infrared spectroscopy of ***para*-TMPyP** revealed (KBr; *ṽ*/cm^−1^): 3327 w (ν_N−H_), 3043 w (ν _= C−H_), 2618 w, 1458 w (δ_C−H_) 1189 s ($\nu_{{\mathrm{SO}}_{2}}$ ), 1036 s (ν_S = O_), 1013 m, 803 s (γ _= C−H_) 684 m, 561 m.

Therefore, our goal was to investigate the structure–activity relationship of porphyrinoid PSs in more detail. Here, we focused on the tetracationic PS TMPyP [30,32,55,56,57] and the possible influence of its different arene substitution patterns on photodynamic inactivation potential. The relative positions of substituents in aromatic hydrocarbon backbones are designated by the substitution patterns as part of chemical nomenclature. In organic chemistry, the Greek prefixes *meta*, *para,* and *ortho* refer to the position of the second substituent in relation to the porphyrin backbone. While the widely available and investigated form of TMPyP is substituted with four positively charged *N*-methylpyridinium groups in *para* position (***para*-TMPyP**), we synthesized and characterized the *meta*-substituted variant ***meta*-TMPyP** (Figure 1). 

In both TMPyP isomers, the C_meso_−C_pyr_ bonds are freely rotational, but the position of the *N*-methylpyridinium group changes in relation to the porphyrin backbone and can vary in ***meta*-TMPyP**, as it possesses more degrees of freedom than the ***para*-TMPyP**. Differences in the photophysical properties of these two isomeric tetrakis(N-methylpyridiniumyl) porphyrins have been described earlier [58], especially with regard to absorption spectral features. The Q(0,0) as well as the Soret band has maximum intensity in the *meta* isomer and decreases as one goes through the series *meta* > *para* > *ortho*.

Moreover, the different position of the *N*-methylpyridinium group might result in differences in the interactions with cell components. It is also conceivable that the free rotatability displaces reactive oxygen species (ROS) that are formed on ***meta*-TMPyP** and thus decomposition occurs less quickly in comparison to ***para*-TMPyP**. Other possible differences with consequences for PDI effectivity include solubility, lipophilicity, phototoxicity, and tendency for self-aggregation.

Thus, we hypothesized a superior effect of PDI based on ***meta*-TMPyP** in comparison the widely studied ***para*-TMPyP**.

## 2. Materials and Methods

### 2.1. Synthesis of Photosensitizers

All reactions were carried out under an argon atmosphere using standard Schlenk techniques. *N,N*-Dimethylformamide (DMF) was freshly dried over CaH_2_ and distilled prior to use. Methyl *p*-toluenesulfonate was obtained from Fischer Scientific GmbH in 98% purity and was used as received. 5,10,15,20-tetrakis(3-pyridyl)porphyrin (*meta*-TPyP) and 5,10,15,20-tetrakis(4-pyridyl)porphyrin (*para*-TPyP) were obtained from PorphyChem SAS in 97% purity and were used without further purification.

For TMPyP variant synthesis, the respective TPyP variant (0.4 g, 0.6 mmol) was stirred at 60 °C in dry DMF (100 mL) until all solids were dissolved. Methyl *p*-toluenesulfonate (17.81 g, 95.7 mmol, 100 eq.) was added in one portion and the resulting reaction mixture was refluxed during 18 h in darkness. After slowly cooling to room temperature, the volume of DMF was reduced to 5 mL under vacuum. The product was precipitated by addition of acetone (100 mL) under stirring. The resulting purple precipitate was separated by filtration, washed thoroughly with acetone (3 × 50 mL) and dissolved in water (100 mL). The aqueous phase was washed with dichloromethane (5 × 200 mL) and concentrated to 5 mL under vacuum. After addition of acetone (100 mL) under stirring a precipitate was formed, which was separated by filtration and thoroughly washed with acetone (3 × 50 mL). The product was obtained after drying under vacuum. 

### 2.2. Compound Analysis

^1^H NMR (500.3 MHz) and ^13^C{^1^H} NMR (125.7 MHz) spectra were recorded with a Bruker Avance III 500 spectrometer (Bruker Corp., Billerica, MA, USA) operating at 293 K in the Fourier transform mode. Chemical shifts are reported in *δ* units (ppm) downfield from tetramethylsilane with the solvent as the reference signal (^1^H NMR, DMSO-d_6_, *δ* 2.50 ppm; ^13^C{^1^H} NMR, DMSO-d_6_, *δ* 39.52 ppm). 

UV–vis absorption spectra were measured with a GENESYS 6 UV–visible spectrophotometer (Thermo Fisher Scientific, Waltham, MA, USA) in the range of 300 to 800 nm at room temperature. Samples of 200 µL with 10 μM solutions of the respective porphyrins in 0.9% NaCl solution were irradiated in air under the same conditions used for the PDI experiments (i.e., wavelength, light dose, layer thickness, temperature). Electronic absorption spectra after indicated irradiation times were recorded. The stability of the respective porphyrin was determined by the decay in the absorbance of their most intense absorption band (B band) over irradiation time.

FT-IR spectra were recorded with a Thermo Nicolet IR 200 spectrometer (Thermo Fisher Scientific) at room temperature as KBr pellets. LC–MS analyses of ***meta*-TMPyP** were performed on a timsTOF mass spectrometer (Bruker Corp.) equipped with an ESI source. Samples were prepared by diluting aqueous stock solutions of the analytes (1 mg ∙mL^−1^) by a factor of 1:100 with a mixture of water/acetonitrile (V/V, 1:1) and measured at flow rates of 10 μL min^−1^.

Crystallographic data of **[*meta*-TMPyP][Tosylate]_4_Me⋅OH** were collected with a Rigaku Oxford Gemini S diffractometer at 100 K. The structure was solved by direct methods and refined by full-matrix least-square procedures on F^2^ [59]. All non-hydrogen atoms were refined anisotropically and all hydrogen atoms using riding models. The asymmetric unit comprised a disordered MeOH molecule with an overall occupation factor of 0.5 and split occupancies of 0.335/0.165. The CCDC deposit 2162884 contains full experimental detail of the SCXRD study and of all bond lengths and bond and torsion angles (www.ccdc.cam.ac.uk/structures; deposited on 29 March 2022).

### 2.3. Bacterial Strains

For the PDI experiments, clinical strains of extended spectrum *β*-lactamase (ESBL)-producing and fluoroquinolone-resistant *Escherichia coli* (*E. coli)* were used. Minimum inhibitory concentrations were determined using the ISO 20776-1 microbroth dilution method. Phenotypic ESBL production was confirmed using the Etest (bioMérieux, Marcy l’Etoile, France). Antibiotic susceptibilities were assessed using the clinical breakpoints (Version 9.0) established by the European Committee on Antimicrobial Susceptibility Testing in 2019.

For preparation of bacterial suspensions, bacteria were cultured aerobically overnight at 37 °C on blood agar (Carl Roth, Karlsruhe, Germany). Then, colonies were collected with a sterile cotton bud (Peha^®^, Hartmann, Heidenheim, Germany) and diluted in 0.9% NaCl to an initial bacterial suspension of McFarland standard No. 1 (approx. cell density 3.0 × 10^8^ CFU/mL). 

To control for the final bacterial density, the prepared suspensions, diluted 1:10, 1:100 and 1:10000, were inoculated on blood agar plates, incubated for 36 h at 37 °C in the dark. Subsequently, colony-forming units (CFU) per ml were counted on plates with the most appropriate dilution.

### 2.4. Photosensitizers

The two water-soluble PSs 3,3′,3″,3‴-(5,10,15,20-tetrayl)tetrakis(1-methylpyridin-1-ium)porphyrin tetra-4-methylbenzenesulfonate and 4,4′,4″,4‴-(5,10,15,20-tetrayl)tetrakis(1-methylpyridin-1-ium)porphyrin tetra-4-methylbenzenesulfonate, denoted in the following as ***meta*-TMPyP** and ***para*-TMPyP**, were synthetized as described above. Stock solutions of both compounds with concentrations of 800 µM in 0.9% NaCl were prepared and stored in the dark at 4 °C for up to one week prior to use.

### 2.5. Photodynamic Inactivation Procedure

For PDI experiments, bacterial suspension at indicated density was pipetted into sterile 96-well plates with clear F-bottom (Greiner Bio-One, Frickenhausen, Germany) at a volume of 100 µL per sample. Then, ***meta*-TMPyP** and ***para*-TMPyP** were added (100 µL; 800 µM) and after careful mixing for 30 s and an overall preincubation period of 4 min in the dark, the samples were illuminated with LED. The final concentrations of ***meta*-TMPyP** and ***para*-TMPyP** were set to 400 µM. Illumination duration was set to 36 to 72 min for LED light application (up to 108 min for light-only controls). 

A custom-made 3 by 4 LED array (High Power LED from Avonec^®^, Wesel, Germany) with the required wavelength (*λ* = 420) nm for PS excitation was used for homogenous illumination of the samples, as previously described [60]. The light intensity was relatively constant (>85% of peak intensity) within an area of 10 × 14 cm^2^ and at a distance of 8 cm between LED and sample. A power supply (Mean Well HLG-40H-54A, 40 W 0.75 A 54 V/DC PFC, Mean Well Enterprises Taiwan) was used to provide 40 W at 54 V to the LED array. At this setting, the total optical power applied to the samples was 11 W at a power density of 13 mW/cm^2^ at the sample surface.

Additional samples were prepared as dark toxicity controls. These were performed with the same test setup including preincubation with ***meta*-TMPyP** and ***para*-TMPyP**, but without illumination. Further controls were performed using vehicle (0.9% NaCl) without PS, followed by standard illumination treatment (light controls). Subsequently, all treated samples were cultured on blood agar culture plates for determination of density as described above. All experimental conditions were performed at least in triplicate (as indicated).

### 2.6. Detection of Singlet Oxygen

The generation of singlet oxygen was analyzed using the fluorescent dye, 9,10-anthracenediyl-bis(methylene)dimalonic acid (ABDA), as described previously [44]. ABDA reacts selectively with singlet oxygen to a nonfluorescent endo-peroxide product. For this test, 250 µL of a solution containing 400 µM ABDA and 10 µM ***meta*-TMPyP** or ***para*-TMPyP** was injected in 96-well microtiter plates. The samples were illuminated as described above. The fluorescence of the samples was measured photometrically every 2 min using an infinite M200 pro microtiter plate reader from Tecan (Maennedorf, Switzerland). The fluorescence maximum of ABDA at 422 nm (excitation at 378 nm) was used for analysis. As reference (light control) a solution containing 400 µM ABDA but no ***meta*-TMPyP** or ***para*-TMPyP** was used.

### 2.7. Statistical Analysis

Data are expressed as mean ± SD. Differences between groups were tested by one- or two-way ANOVA with Sidak’s post-hoc test where appropriate or Student’s t-test and Bonferroni correction for multiple comparisons. Calculations were performed using the Graph Pad Prism Software version 7.0 (GraphPad Software Inc., La Jolla, CA, USA). *p* < 0.05 was considered indicative for significant differences.

## 3. Results

### 3.1. Synthesis Results

**Figure 2:**
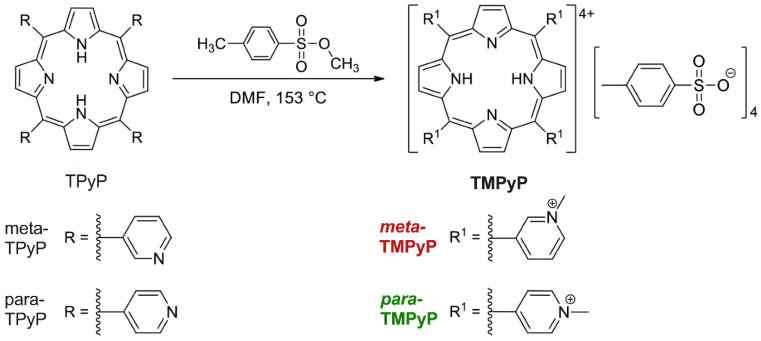
Synthesis reaction scheme of the variants ***meta*-TMPyP** and ***para*-TMPyP** For synthesis, the respective porphyrin (*meta*-TPyP or *para*-TPyP) was heated with the methylation reagent *para*-toluenesulfonic acid methyl ester (methyl tosylate) (100 equivalents, 25-fold excess based on the porphyrins) in the dried solvent *N*,*N*-dimethylformamide (DMF) for 18 h under light exclusion and argon atmosphere under reflux at 153 °C. Purification was performed by precipitating the product with acetone and washing an aqueous solution of the product with dichloromethane. The ***meta*-TMPyP** product was obtained in 87% yield as a purple solid, while synthesis of ***para*-TMPyP** yielded 85% of the product as a purple solids.

The synthesis of ***meta*-TMPyP** and of ***para*-TMPyP** was made as described above in the Material and Methods section and as depicted in Figure 2. In this work DMF was applied, whereby reaction solutions of variants of TPyP were refluxed for 18 h in the presence of a 25-fold excess of methyl tosylate. Both ***meta*-TMPyP** and ***para*-TMPyP** were obtained as purple solids in 87 and 85% yield, respectively. Selected data of the ^1^H-NMR and ^13^C{^1^H}-NMR as well as of IR spectroscopic measurements of both products are displayed in the figure caption of Figure 1. All data are in full agreement with the assumption of tetra-methylated products. However, an LC–MS analysis using a timsTOF mass spectrometer revealed that a fraction of the obtained ***meta*-TMPyP** was not fully methylated but a mixture of tetra-, tri-, and di-methylated species was obtained in a 768:88:1 molar ratio (Figure S1).

### 3.2. Single Crystal X-ray Crystallographic Diffraction Study

Crystallization of ***meta*-TMPyP** from highly diluted MeOH solution by slow diffusion of THF resulted in crystals suitable for a single crystal X-ray crystallographic diffraction study (SCXRD). Of note, the formation of amorphous thin films on the glass walls was observed, which is attributed to the precipitation of the tri- and/or di-methylated species. Using more highly concentrated MeOH solutions gave rise to rather amorphous and/or microcrystalline materials. The SCXRD study confirmed the composition of the single crystals as **[*meta*-TMPyP][Tosylate]_4_⋅MeOH.**

Table S1 states selected bond lengths and angles of the **[*meta*-TMPyP]^4+^** fragment in comparison with data reported for ***meta*-TPyP**, while Figure S2 displays the molecular structure of the **[*meta*-TMPyP]^4+^** fragment and Figure S3 illustrates a selected part of one of the chains formed by the **[*meta*-TMPyP]^4+^** fragment in the solid state. 

The solvent adduct **[*meta*-TMPyP][Tosylate]_4_⋅MeOH** with its **[*meta*-TMPyP]^4+^** fragment crystallized in the centrosymmetric space group P-1 and thus possessed crystallographically imposed *C*_i_ symmetry with the inversion center located in the middle of the atoms N1, N2, N1A, and N2A (Figure S2). 

As observed for ***meta*-TPyP [61]** the **[*meta*-TMPyP]^4+^** fragment was also found in the ααββ conformation in the solid state. The dihedral angles between a calculated mean plane of the C_20_N_4_ porphyrin macrocycle and the pyridyl rings were 71.4(1)° and 73.6(1)° (Figure S4) and thus substantially larger compared to the ones of ***meta*-TPyP** for which 62.63(7)° and 64.63(6)° have been reported [61]. On the contrary, for ***meta*-TPyP** a slight wave distortion of the porphyrin macrocycle was observed (r.m.s. deviation from planarity of 0.048 Å) [59,62], whereby the macrocycle of the **[*meta*-TMPyP]^4+^** fragment was essentially flat (r.m.s. deviation from planarity of 0.028 Å, Figure S4). 

Interestingly, molecules of tetracationic **[*meta*-TMPyP]^4+^** were packed in the same manner as the ones of ***meta*-TPyP** in the typical off-set style into chains with cofacial aromatic interactions (Figure S3). As shown in Table S1, related bond lengths and angles can also be regarded as analogous. 

### 3.3. UV–VIS Analysis

The absorption spectra in the range of 300 to 700 nm of ***meta*-TMPyP** and ***para*-TMPyP** in aqueous solutions at room temperature are shown in Figure S5. Both compounds exhibited characteristic free base porphyrin absorption spectra with an intense Soret (or B) band maximizing at 418 nm (***meta*-TMPyP**) and at 422 nm (***para*-TMPyP**), respectively, together with four less intense Q bands in the range from 515 nm to 636 nm (***meta*-TMPyP**) and 518 nm to 643 nm (***para*-TMPyP**), respectively (Table 1 and Figure S5). A slight hypsochromic shift (left shift to blue light) was observed for the absorption maxima of ***meta*-TMPyP** as compared to ***para*-TMPyP**. Thus, the isomerism at the *meso*-pyridinium groups had a small but significant influence on both position and shape of the UV–VIS absorption bands.

The B bands are particularly interesting for PDI applications, because they have the highest molar extinction coefficients of both porphyrins. Hence it could be assumed that excitation at the B bands would lead to the highest achievable yields of ^1^O_2_ and/or other ROS. The molar extinction coefficient of the B band of ***meta*-TMPyP** (*ε* = 301995 dm^3^∙mol^−1^∙cm^−1^) exceeded that of ***para*-TMPyP** (*ε* = 194,984 dm^3^∙mol^−1^∙cm^−1^) by about 55%. Thus, a more intense formation of ^1^O_2_ could be expected for ***meta*-TMPyP**. Furthermore, no concentration-induced shifts in the position of the absorption maxima were observed (Figure S6), which excluded concentration-dependent self-aggregation within the investigated concentration range [63].

### 3.4. Photostability

Given their efficient production of ROS upon illumination, many porphyrins are prone to photooxidation which can significantly decrease their photodynamic activity due to the decomposition of their chromophoric system [64,65,66]. To determine the influence of such photodegradation on the PDI experiments described herein, the photostability of ***meta*-TMPyP** and ***para*-TMPyP** in aqueous solutions was investigated. 

**Figure 3:**
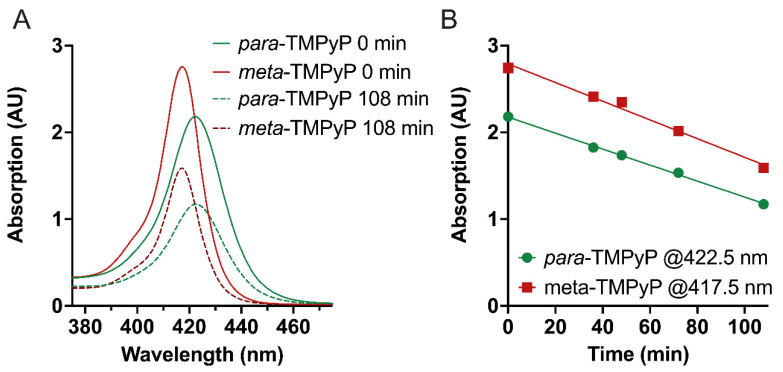
Photostability of ***meta*-TMPyP** and ***para*-TMPyP** as determined by measurements of absorption after illumination at 420 nm LED light with an intensity of 13 mW/cm^2^ for indicated durations (0.78 J/cm^2^ per minute). (**A**) UV–VIS absorption spectra in the range of their most intense absorption band (B band) before and after 108 min of illumination. Please note the hypsochromic shift of the absorption maximum by 4 nm as well as the stronger absorption of the **meta-TMPyP** variant when compared to ***para*-TMPyP** (see Table 1). (**B**) Change in absorption at the respective wavelengths and indicated time points during illumination. An approximately linear decay of absorption was observed for both TMPyP variants.

Both porphyrins exhibited an approximately linear decay in concentration over irradiation time (Figure 3 and Figure S7). 

The maximum irradiation time was set to 108 min, as the PDI experiments were performed with the same illumination time. After 108 min about 58% of ***meta*-TMPyP** and about 54% of ***para*-TMPyP** were still present and no new bands were observed in the absorption spectra between 350 nm and 750 nm. Hence, the different isomeric substitution pattern of the pyridinium groups had only a small influence (+6.9% ***meta*-TMPyP**) on the photostability. 

### 3.5. Generation of Singlet Oxygen

**Figure 4:**
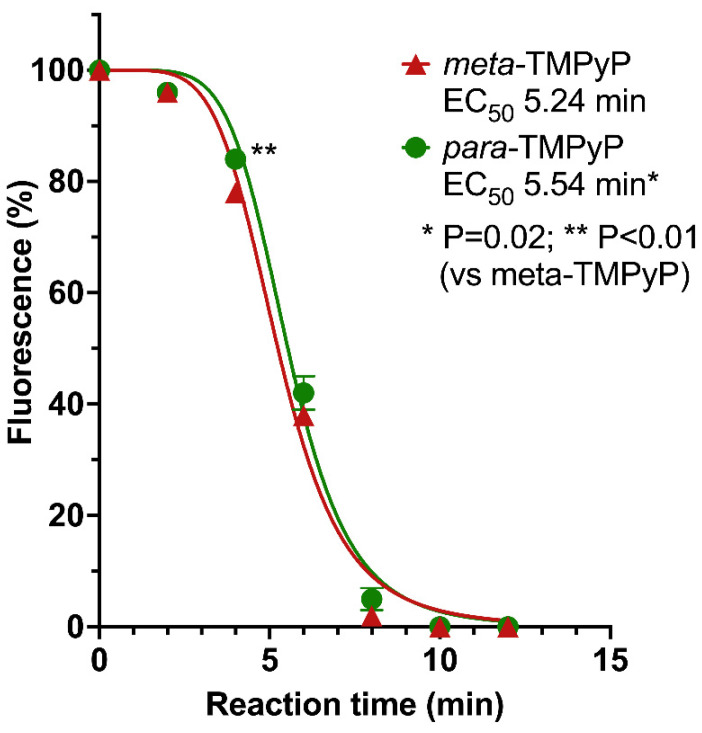
Generation of singlet oxygen by ***meta*-TMPyP** and ***para*-TMPyP** as detected by ABDA reaction test. Both TMPyP variants were exposed to LED light under the same conditions as in PDI experiments (0.78 J/cm^2^ per minute). Generation of singlet oxygen time-dependently led to a reduced fluorescence of remaining ABDA substrate. For ***meta*-TMPyP**, the half-maximal reduction of fluorescence was calculated to be reached only slightly earlier (18 s) than for ***para*-TMPyP**, with a small, but significant, difference in fluorescence detected only at 4 min reaction time.

To determine the effects of the different isomeric substitution patterns of the pyridinium groups on in vitro singlet oxygen production, which was conceivable based on the identified differences in B band absorption coefficients, hypsochromic shift and photostability, we performed the ABDA test as previously described [44,45]. For ***meta*-TMPyP**, the half-maximal reduction of fluorescence (EC_50_) was calculated to be reached only slightly earlier (18 s) than for ***para*-TMPyP** (5.24 vs. 5.54 min, *p* = 0.02), with a small, but significant difference in fluorescence detected only at 4 min reaction time (Figure 4, *p* < 0.01). Based on these results, one would expect only approximately 5.4% more effective singlet oxygen production of ***meta*-TMPyP**.

### 3.6. Photodynamic Experiments

**Figure 5:**
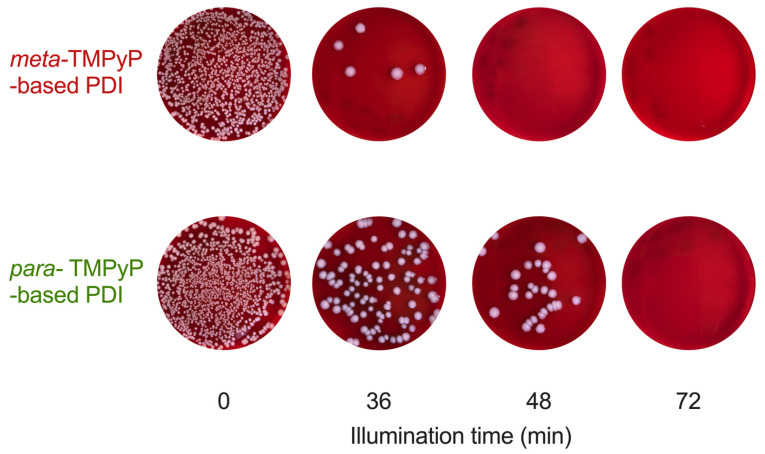
Photographic representation of multidrug-resistant *Escherichia coli* cultures after photodynamic inactivation with ***meta*-TMPyP** versus ***para*-TMPyP**. After treatment with photosensitizer (PS) only without illumination (400 µM; 0 min), no reduction of bacterial density was observed, while cultures treated with photodynamic inactivation (PS and light application; PDI; *n* = 3) showed time-dependent reduction of bacterial growth (36–72 min of illumination; light dose 28–56 J/cm^2^). Please note that application of ***meta*-TMPyP** led to more pronounced effects than ***para*-TMPyP**.

Typical results from PDI experiments as performed with both TMPyP variants under the same conditions (400 µM PS concentration, 4 min preincubation, LED light with maximum intensity at 420 nm) and subsequent colony counting on agar culture plates are depicted in Figure 5. After treatment with the PS only without illumination (0 min), no reduction of bacterial density was observed, indicating the absence of relevant PS toxicity. Similarly, no relevant changes in bacterial density were observed when performing illumination without PS (8.44 × 10^7^ CFU/mL at 0 min, 7.54 × 10^7^ CFU/mL at 4 min, 8.75 × 10^7^ CFU/mL at 12 min, 8.49 × 10^7^ CFU/mL at 36 min, 8.31 × 10^7^ CFU/mL at 108 min; *n* = 6; *p* = 0.87). 

**Figure 6:**
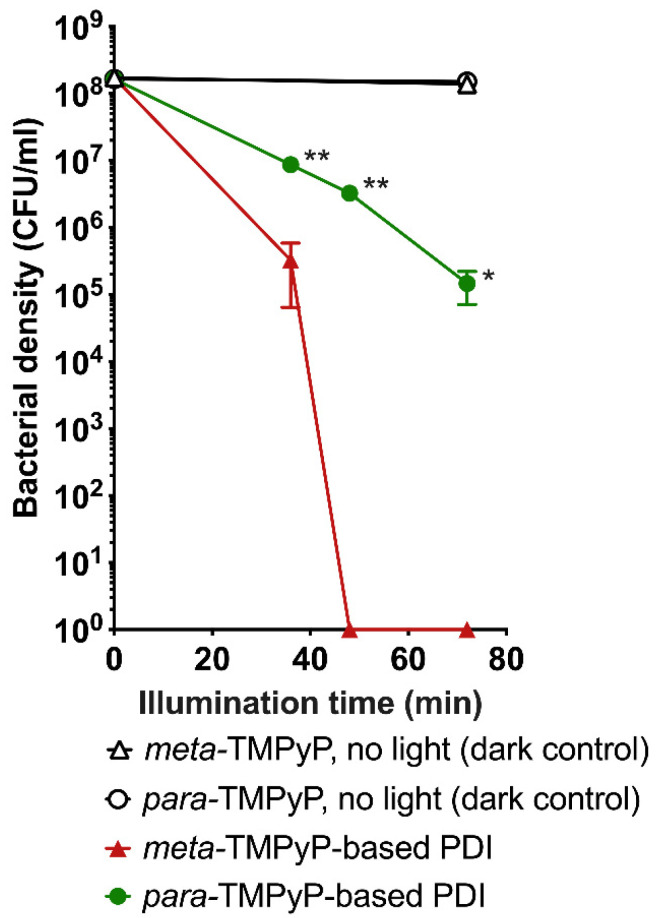
Dose-dependent effects of LED-based photodynamic inactivation based on ***meta*-TMPyP** in comparison to ***para*-TMPyP**. Increasing durations of light illumination (illumination dose) during the PDI experiment with ***meta*-TMPyP** or ***para*-TMPyP** (400 µM) led to a reduction of bacterial density in multidrug-resistant *Escherichia coli*. The effects of ***meta*-TMPyP**-based PDI were significantly stronger after illumination times of 36, 48, and 72 min (* *p* < 0.05; ** *p* < 0.01; light dose 28–56 J/cm^2^) compared to ***para*-TMPyP**. Dark controls without illumination showed no reduction of bacterial density. Data are represented as mean ± SD.

Photodynamic inactivation (PS and light application; PDI; *n* = 3) of ESBL-producing and fluoroquinolone-resistant *E. coli* resulted in a time-dependent reduction of bacterial growth (36 to 72 min of illumination; Figure 5 and Figure 6). Please note that application of ***meta*-TMPyP** led to more pronounced effects than ***para*-TMPyP**, reaching a sterilizing effect after 48 min illumination duration (*p* < 0.01).

## 4. Discussion

Our results confirm the previously reported antimicrobial effects of TMPyP-based photodynamic inactivation of ESBL-producing *E. coli* in vitro [33,40,41,56]. Overall, porphyrinoid-based PS are considered as non-toxic and well-tolerated at concentrations that are required for antimicrobial PDT [67]. Likewise, we observed no significant dark toxicity of ***meta*-TMPyP** in this investigation.

With this report, we extend the evidence for TMPyP-based antimicrobial PDT in several aspects beyond the findings from earlier studies: As hypothesized, there was a difference in inactivation efficacy between the commonly used ***para*-TMPyP** and the differently substituted ***meta*-TMPyP**. While there were only minor differences with regard to photostability and luminous efficacy of singlet oxygen generation, we found a drastically improved antimicrobial action of PDI when based on ***meta*-TMPyP**. 

With regard to our crystallographic analysis, one has to keep in mind that the choice of the reaction conditions significantly determines the degree of alkylation and therefore we chose DMF. The first report on the alkylation of variants of TPyP from 1982 [68] already made use of DMF as the reaction solvent as did a very recent report on the synthesis of ***meta*-TMPyP** and of ***para*-TMPyP** [69], while further synthetic conditions were unfortunately not given.

In order to verify whether or not **[*meta*-TMPyP][Tosylate]_4_⋅MeOH** forms aggregates in aqueous solutions as the solvent used for PDI studies, we attempted to crystallize it from such solutions. All trials gave, however, microcrystalline materials only. Nevertheless, an SCXRD study of **[*para*-TMPyP][Tosylate]_4_⋅2H_2_O [70],** crystallized from an aqueous solution equilibrated against pentane-1,2-diol, revealed the tetracationic **[*para*-TMPyP]^4+^** fragments as not involved in intermolecular interactions with each other but as fully surrounded by both the **[Tosylate]^-^** anions and the water molecules. 

Recently, we reported on the use of 3,3′,3″,3‴-(7,8,17,18-tetrahydro-21H,23H-porphyrine-5,10,15,20-tetrayl)tetrakis [1-methyl-pyridinium]tetratosylate (**THPTS**) as an effective PS for PDI of critical multidrug-resistant bacteria [47]. The molecular and crystal structure of **THPTS** was reported as well and it was shown that this compound is, as **[*para*-TMPyP][Tosylate]_4_⋅2H_2_O** [62], in the solid state fully surrounded by the **[Tosylate]^-^** anions [47]. Based upon these observations, it therefore seems very likely that molecules of tetracationic **[*meta*-TMPyP]^4+^** are not involved in aqueous solution in intermolecular interactions with each other. A further confirmation of this assumption was derived from UV–VIS spectroscopic measurements of **[*meta*-TMPyP][Tosylate]_4_**. After irradiation, no new bands were observed in the absorption spectra between 350 nm and 750 nm. Hence, the decomposition products cannot be electronically excited in this range and therefore do not act as PSs and/or potentially photosensitizing material.

Compared to the literature, the UV–VIS spectroscopic data of TMPyP are in good agreement [69,70]. However, the illumination-dependent decomposition occurred more rapidly in our measurements than reported previously. In another study, much higher decay times for ***meta*-TMPyP** and ***para*-TMPyP** were estimated [69]. From our point of view, the higher stability in their measurements was due to irradiation at 365 nm for both ***meta*-TMPyP** and ***para*-TMPyP**, where both PSs possessed a dramatically lower extinction compared to 420 nm, as used in this study (Figure 3 and Figure S5). Additionally, they used a lower light dose [69].

Previously, TMPyP and similar porphyrins were found to be very effective for inactivating Gram-positive bacteria and fungi even at low nanomolar concentrations, whereas Gram-negative bacteria were much less susceptible [71]. Structural factors affecting the subcellular localization of the PS are associated with the total charge (which ranges from −4 to +4 for tetraphenyl derivatives), the octanol–water partition coefficient, and the substitution pattern at the tetrapyrrole ring [72,73]. Current research regarding optimization of PSs for antibacterial PDI therefore focuses on charged side groups, which connect to a bacterial wall through electrostatic interactions [74,75]. For instance, positively charged porphyrins interact strongly and accumulate in Gram-negative bacteria strains [20,45,46]. In addition, (*N*-methylpyridinium-4-yl)-substituted PSs are also known to directly bind to DNA [76]. Moreover, porphyrin ring modifications can change physicochemical features and modulate redox properties, charge distribution, and lipophilicity [24]. Our analysis has found that ***meta*-TMPyP** is more effective in reducing bacterial density of multidrug-resistant *E. coli* in PDI experiments when compared to ***para*-TMPyP**. We documented an hypsochromic shift in the UV–VIS absorption spectrum, together with a higher extinction coefficient for ***meta*-TMPyP**. Therefore, one might speculate that this leads to an improved yield of singlet oxygen. However, the differences in generation of oxygen species and photostability between ***meta*-TMPyP** and ***para*-TMPyP** were rather small, and there most likely do not fully explain the observed difference in biological action. 

In general, many other factors such as differences in overall charge and octanol–water partition coefficients, self-aggregation, and different degrees of freedom for accumulation in bacteria or at bacterial membranes as well as interaction with target structures like bacterial DNA may be considered [77]. Based on our analysis, substantial differences in the proportion of tetracationic molecules and therefore overall charge in the investigated synthesis products for ***meta*-TMPyP** and ***para*-TMPyP** are very unlikely. However, with regard to distribution of the charge, porphyrins with peripheral charged groups in the *meta* position exhibit an asymmetric distribution of the charge around the molecule and therefore an amphiphilic character, leading to a better accumulation in cells [52].

Usually, *meta*-isomers exhibit lower octanol–water partition coefficients than para-isomers, because the out-of-plane arrangement of positively charged peripheral groups leads to a higher dipole moment [78]. Therefore, if there was a relevant difference in the octanol–water partition coefficients of ***meta*-TMPyP** and ***para*-TMPyP**, it would most likely be in favor of ***para*-TMPyP** accumulation. 

Water-soluble porphyrins may form a variety of molecular complexes in aqueous solution through noncovalent interactions [79]. However, TMPyP does not aggregate to a relevant degree in water even in solution with inorganic salts [80]. Our data from UV–VIS analysis of ***para*-TMPyP** and ***meta*-TMPyP** revealed no concentration-induced shifts in the position of the absorption maxima, which excludes concentration-dependent self-aggregation within the investigated concentration range for both variants. Furthermore, it does not seem plausible to assume relevant self-aggregation of ***meta*-TMPyP** in water with its H-bridges and its high dipole moment. Based on previously published data, this can also be fully ruled out for ***para*-TMPyP** [80,81]. As reported earlier, the acid–base dissociation constant pK_a_ of ***meta*-TMPyP** is slightly higher (1.8) than that of ***para*-TMPyP** (1.4) [58]. While both pK_a_ values are very low, the proportion of non-ionized/nonpolar ***meta*-TMPyP** should therefore be higher at the physiological pH range, leading to better cellular uptake.

Taken together, the observed minor differences in photostability and generation of singlet oxygen may contribute to the observed differences in PDI effectivity for the two investigated variants of TMPyP, while it remains unclear which other factors may play a role. 

### Limitations

Since we performed in vitro experiments in bacterial suspensions only, based on these data we cannot predict its efficacy in biofilms or the in vivo setting and also cannot rule out potential undesired effects on surrounding tissue of applying PDI with intense light doses of up to 56 J/cm^2^. Moreover, interaction with Quorum-sensing and other virulence mechanisms may not be exposed by *in vitro* experiments.

## 5. Conclusions

In conclusion, we found an increased efficacy of ***meta*-TMPyP**-based PDI in comparison to the previously available ***para*-TMPyP**. In-detail investigation of the influence of the chemical structure properties of porphyrin-based PSs on their effectivity in PDI applications may help to further improve applicability and treatment success.

## Figures and Tables

**Table 1:** Absorption maxima *λ* and molar extinction coefficient (lg *ε*) of the absorption bands in the UV–VIS spectra of ***meta*-TMPyP** and ***para*-TMPyP** in aqueous solution.

	B Band *λ*/nm	Q Bands *λ*/nm			
	B(0,0) (lg *ε*)	Q_X_(1,0) (lg *ε*)	Q_X_(0,0) (lg *ε*)	Q_y_(1,0) (lg *ε*)	Q_y_(0,0) (lg *ε*)
***meta* -TMPyP**	418 (5.48)	515 (4.27)	550 (3.59)	583 (3.82)	636 (2.94)
***para*** **-TMPyP**	422 (5.29)	518 (4.12)	556 (3.73)	586 (3.78)	643 (3.08)

## Data Availability

Data is contained within the article or the Supplementary Materials.

## Supplementary Materials

The following supporting information can be downloaded at: https://www.mdpi.com/article/10.3390/microorganisms10050858/s1, Figure S1: MS spectra obtained by LC–MS (timsTOF) analysis of ***meta*****-TMPyP**; Figure S2: ORTEP diagram (30% probability ellipsoids) of the molecular structure of the [***meta*****-TMPyP**]^4+^ fragment; Figure S3: Ball-and-stick model of a selected part of one of chains formed by the [***meta*****-TMPyP**]^4+^ fragment; Figure S4: Individual deviations (Å) of atoms of the porphyrin macrocyle of [***meta*****-TMPyP**]^4+^ fragment; Figure S5: UV–VIS absorption spectra of ***meta*****-TMPyP** and ***para*****-TMPyP**; Figure S6: UV–VIS absorption spectra of ***meta*****-TMPyP** and ***para*****-TMPyP** at different concentrations in aqueous solution; Figure S7: Changes in UV–VIS spectra of ***meta*****-TMPyP** and ***para*****-TMPyP** in 0.9% NaCl solution upon irradiation centered at 420 nm; Table S1: Selected bond lengths (Å) and angles (°) of *meta*-TPyP in comparison with the [***meta*****-TMPyP**]^4+^ fragment.
