# Supplementary material for: The Meta-Substituted Isomer of TMPyP Enables More Effective Photodynamic Bacterial Inactivation than Para-TMPyP In Vitro
Source: Microorganisms. 2022 Apr 21;10(5):858. doi: 10.3390/microorganisms10050858 (PMC9143678; doi:10.3390/microorganisms10050858)
Supplement: Supplementary file 1 [file microorganisms-10-00858-s001.zip › microorganisms-1685654-supplementary.pdf]

microorganisms

MDPI

Supplementary Materials:

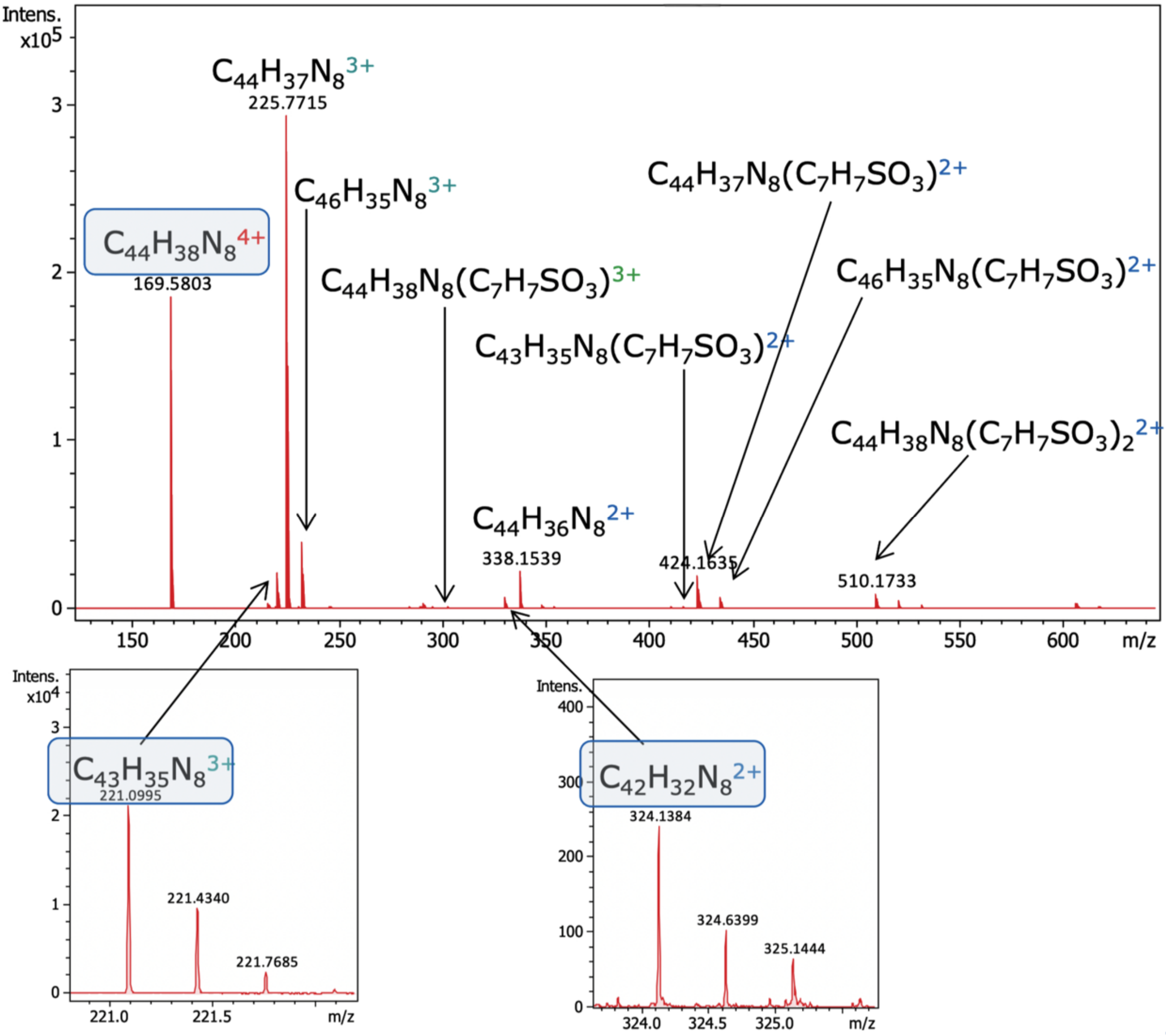

Supplemental Figure S1. MS spectra obtained by LC-MS (timsTOF) analysis of *meta*-TMPyP (obtained as depicted in Figure 1 in DMF**).**

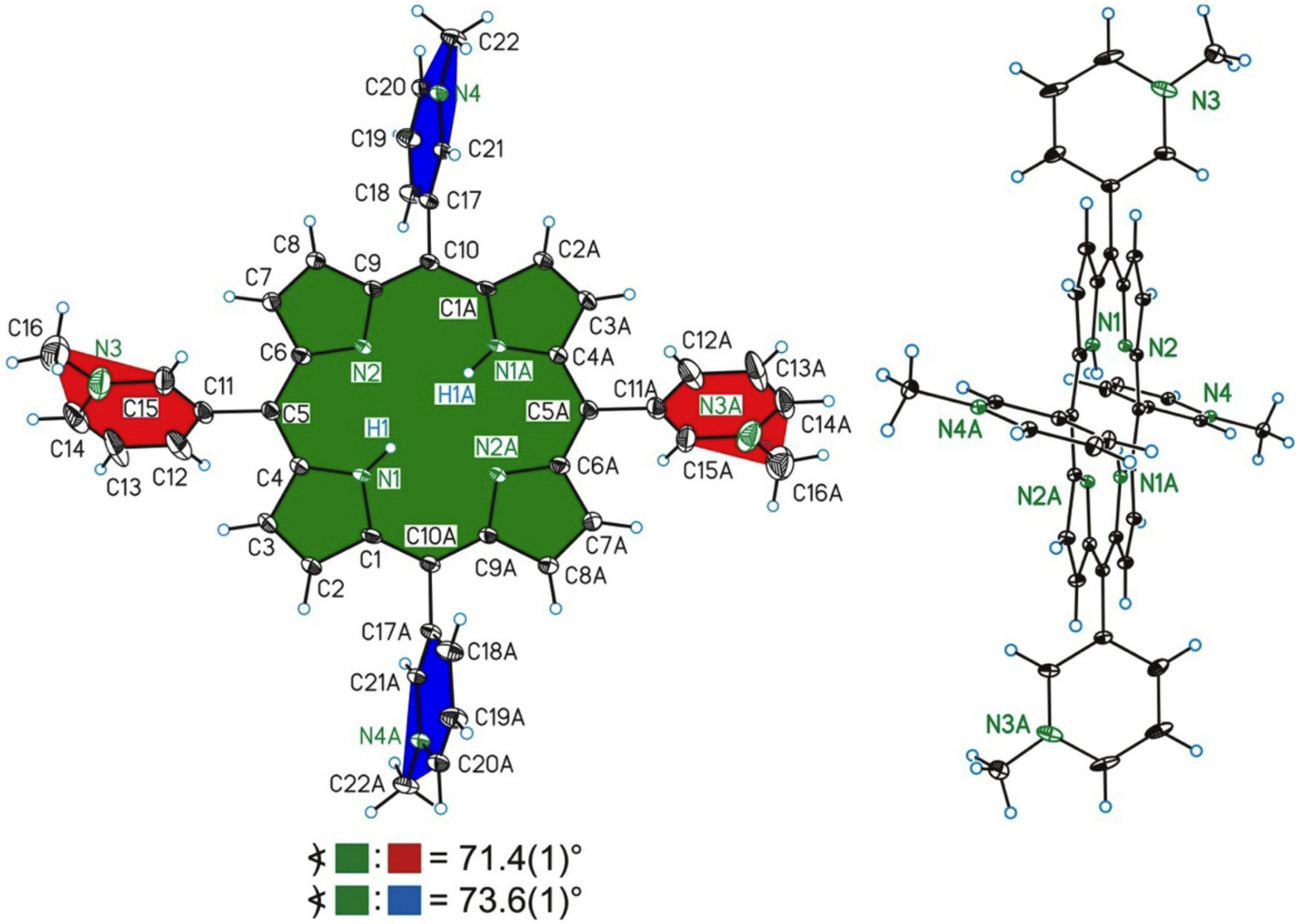

Supplemental Figure S2. ORTEP diagram (30 % probability ellipsoids) of the molecular structure of the [*meta*-TMPyP]$^{4+}$ fragment (in two different perspective views).
Symmetry code: A = -*x* + 2, -*y* + 2, -*z*. The sign ∢ refers to calculated interplanar angles between mean planes of atoms adjoining differently colored functionalities**.**

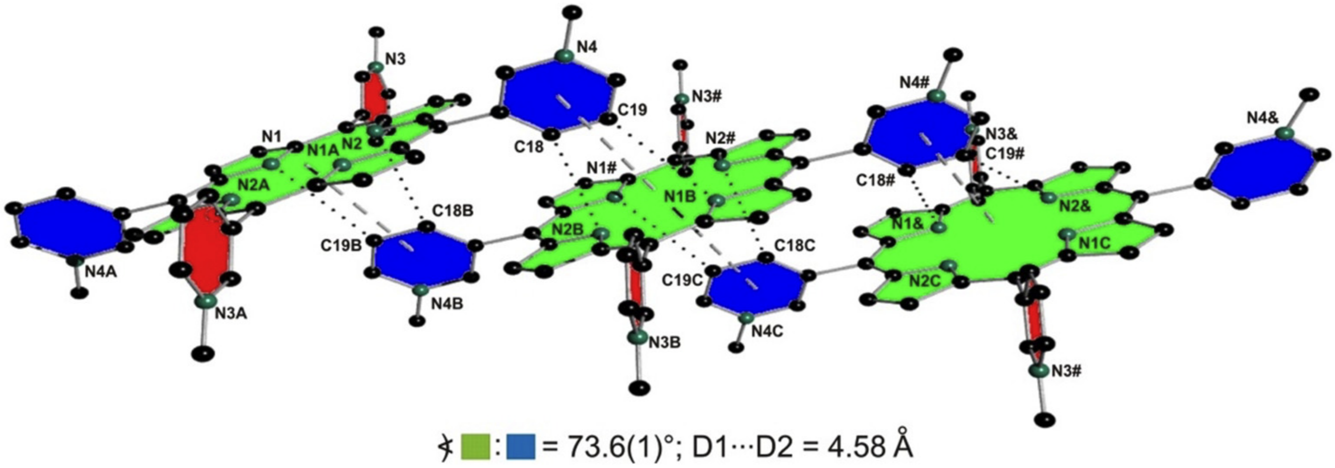

Supplemental Figure S3. Ball-and-stick model of a selected part of one of chains formed by the [*meta*-TMPyP]$^{4+}$ fragment (in the solid state due to intermolecular dispersion type interactions). Symmetry codes: A = 4 − *x*, − *y*, 2 − *z*. B = 3 − *x*, − *y*, 2 − *z*. C = 2 − *x*, − *y*, 2 − *z*. # = *x* − 1, *y*, *z*. & = *x* − 2, *y*, *z*. The sign ∢ refers to calculated interplanar angles between mean planes of atoms adjoining differently colored functionalities. Dashed lines indicate distances between the geometrical centroids of atoms adjoining green (D1) and blue (D2) colored functionalities. Dotted lines refer to the shortest intermolecular distances between molecules of within a chain with *d*(N1···C19B/N2···C18B) = 3.49/3.34 ÅF.

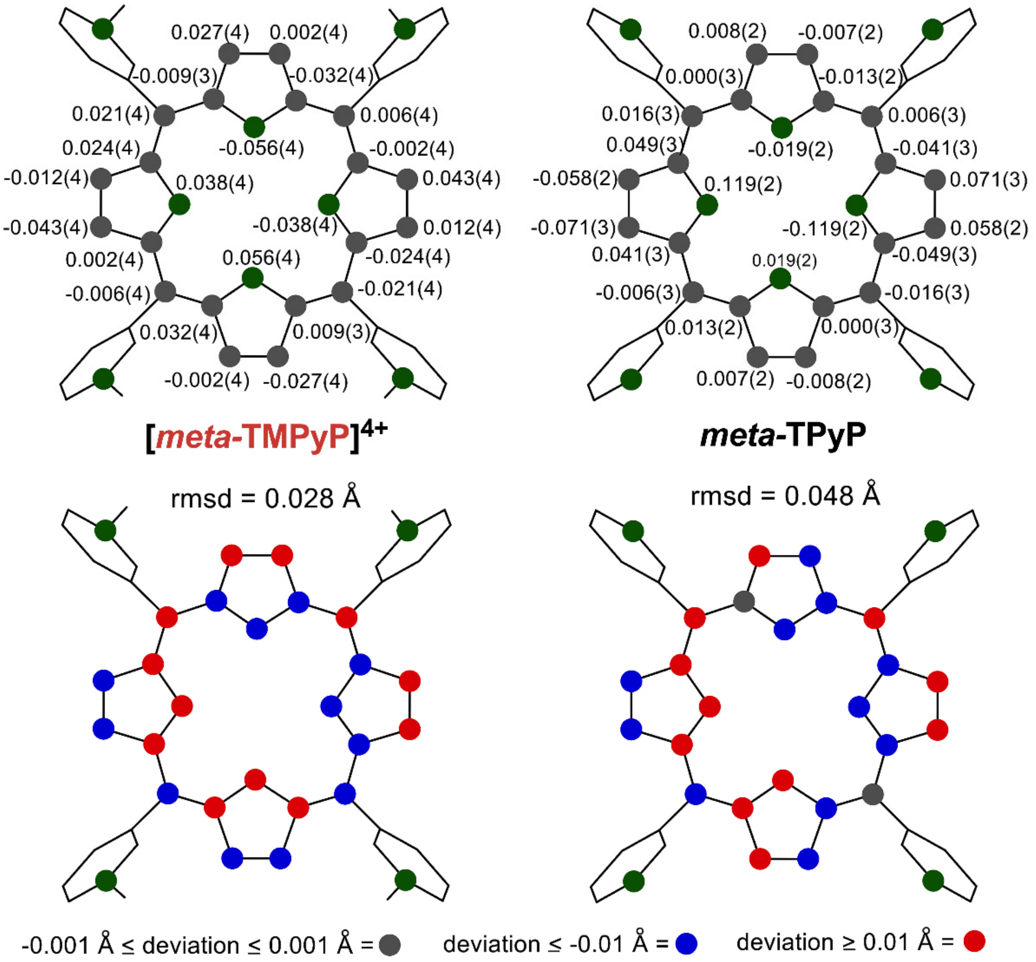

Supplemental Figure S4. Individual deviations (Å) of atoms of the porphyrin macrocycle of [*meta*-TMPyP]$^{4+}$ fragment (in comparison to *meta*-TPyP[S1] from calculated mean planes of all non-hydrogen atoms).

[S1] R. W. Seidel, R. Goddard, C. Hoch, J. Breidung, I. M. Oppel J. Mol. Struct. 2011, 985, 307-315. https://doi.org/10.1016/j.molstruc.2010.11.011

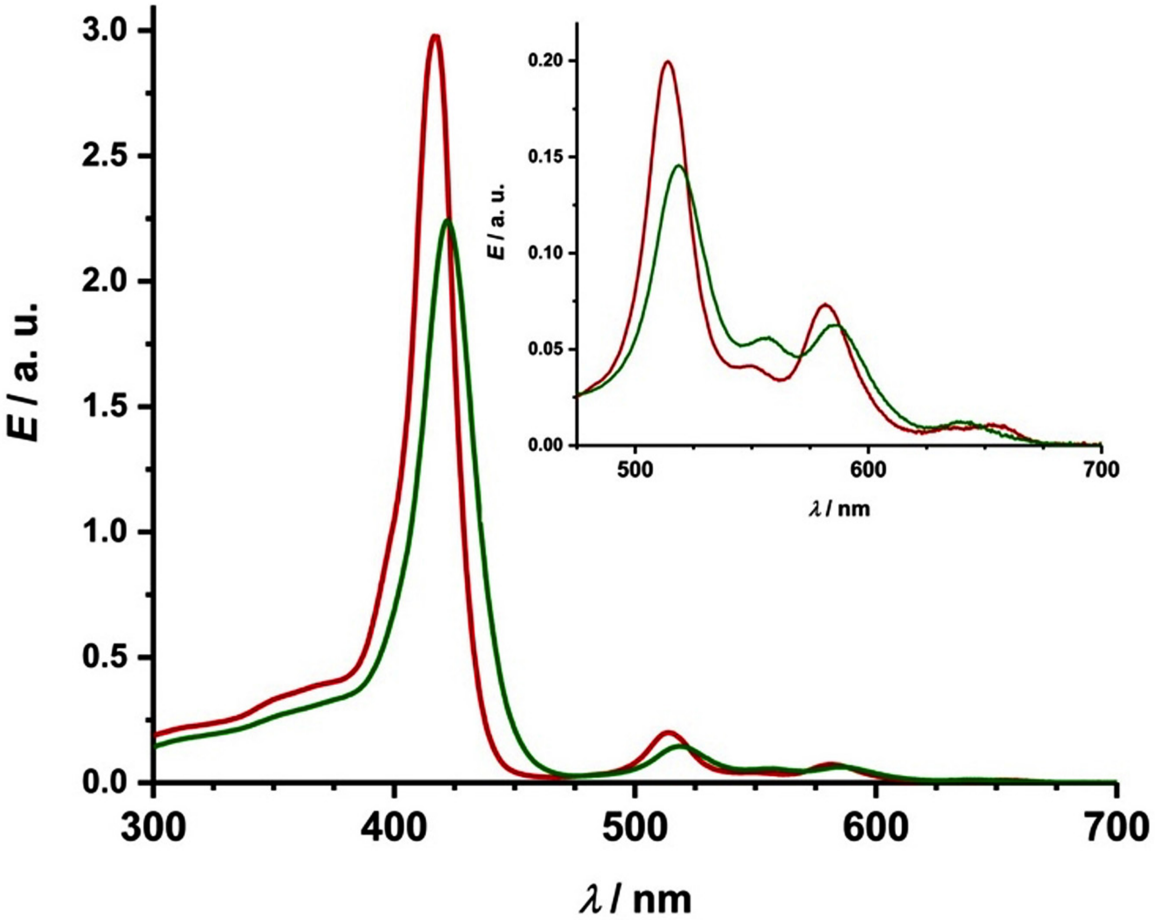

Supplemental Figure S5. UV-vis absorption spectra of *meta*-TMPyP and *para*-TMPyP (in 10 μM aqueous solution at room temperature in the range from 300 to 700 nm). The insert displays the Q bands in an enlarged view. As seen for the B bands, there is a slight hypsochromic shift in absorption maxima as well as higher extinction coefficients found for the ***meta*-TMPyP** variant**.**

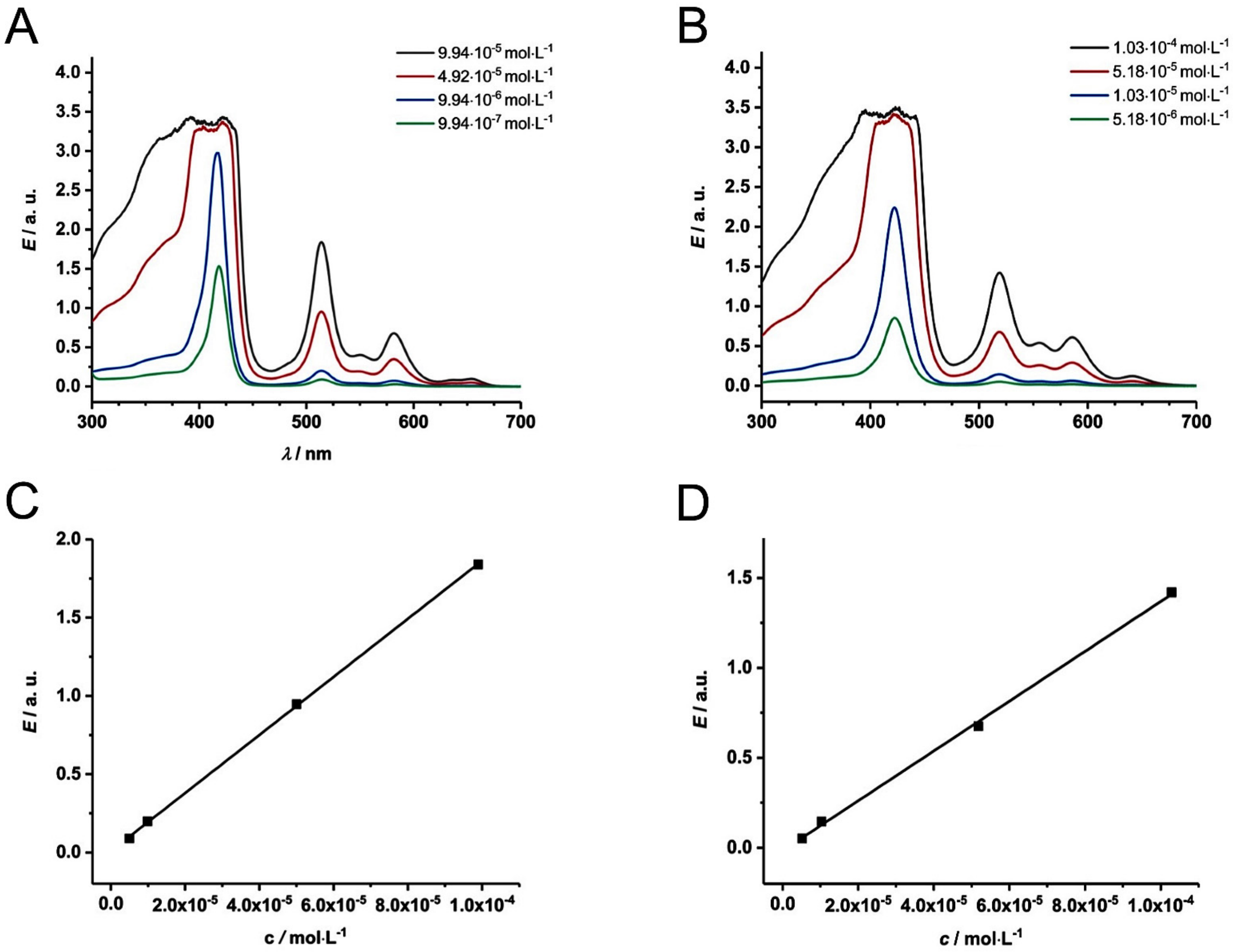

Supplemental Figure S6. UV-vis absorption spectra of *meta*-TMPyP (A) and *para*-TMPyP (B) at different concentrations in aqueous solution. Dependence of the extinction on the concentration of ***meta*-TMPyP** at λ_{max} = 515 nm **(C)** and ***para*-TMPyP (D)**. A linear fit in correspondence with the Beer Lambert law is shown (R^2 = 0.9998 and 0.9990, respectively)**.**

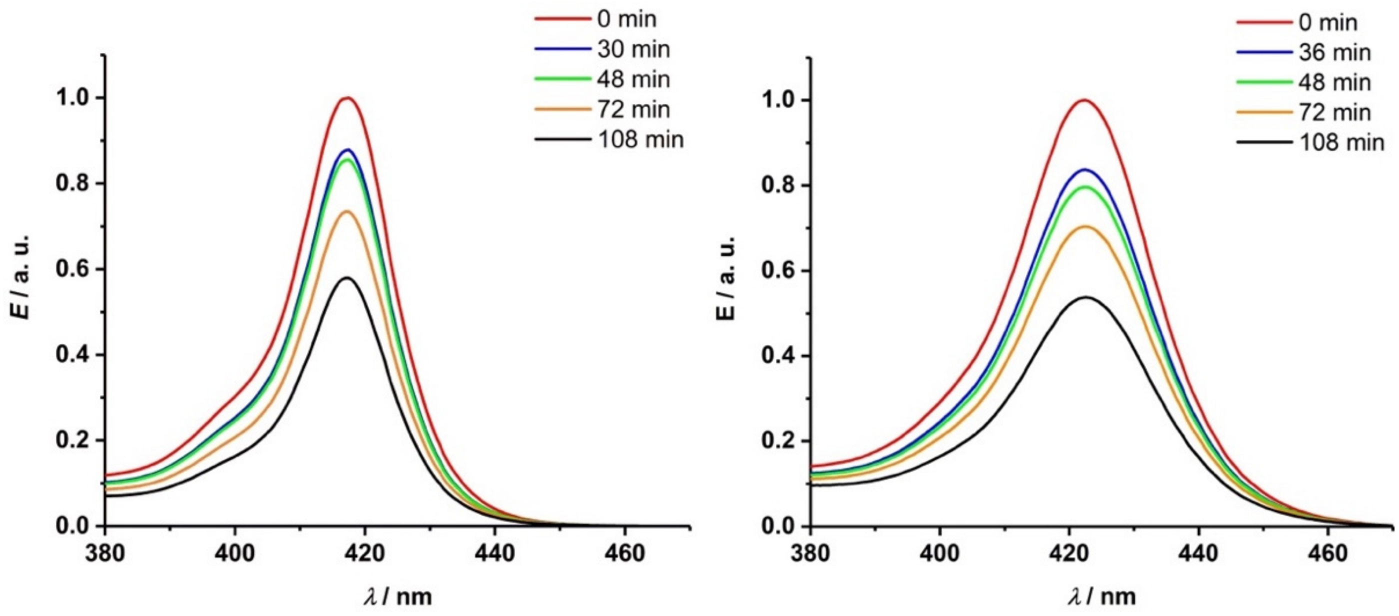

Supplemental Figure S7. Changes in UV-vis spectra of *meta*-TMPyP (left) and *para*-TMPyP (right) in 0.9 % NaCl solution upon irradiation centered at 420 nm. For comparison, the absorbance of the absorption maxima at 0 min were normalized to 1 and all others set accordingly. Please note that no shift in absorption maximum occurred because of illumination treatment and concentration decay**.**

Supplemental Table S1. **Selected bond lengths (Å) and angles (°) of *meta*-TPyP in comparison with the [*meta*-TMPyP]$^{4+}$ fragment.**

a) Symmetry code: A = – x + 2, – y + 2, – z.

Bond length			Bond angles				
	[*meta*-TMPyP]$^{4+}$	*meta*-TPyP		[*meta*-TMPyP]$^{4+}$		*meta*-TPyP	
C1–N1	1.373(5)	1.375(4)	N1–C1–C10A	125.4(4)		126.2(3)	
C1–C10A[a)]	1.395(6)	1.399(4)	N1–C1–C2	108.6(4)	360.0(7)	107.4(3)	360.0(5)
C1–C2	1.441(6)	1.433(4)	C2–C1–C10A[a)]	126.0(4)		126.4(3)	
C2–C3	1.343(6)	1.351(4)	C3–C4–C5	125.4(4)		126.5(3)	
C3–C4	1.441(5)	1.435(4)	C3–C4–N1	108.9(4)	360.0(7)	107.6(3)	360.0(5)
C4–N1	1.360(5)	1.373(4)	N1–C4–C5	125.7(4)		125.9(3)	
C4–C5	1.407(6)	1.401(4)	C4–C5–C6	125.3(4)		126.2(3)	
C5–C11	1.473(5)	1.497(4)	C4–C5–C11	116.4(3)	360.0(6)	116.5(3)	359.9(5)
C5–C6	1.407(5)	1.397(4)	C6–C5–C11	118.3(4)		117.2(3)	
C6–N2	1.362(5)	1.379(4)	C5–C6–N2	125.6(4)		125.7(3)	
C6–C7	1.429(6)	1.446(4)	C5–C6–C7	124.3(4)	359.9(6)	124.5(3)	359.(5)
C7–C8	1.355(6)	1.342(4)	C7–C6–N2	110.0(3)		109.7(3)	
C8–C9	1.442(6)	1.442(4)	C8–C9–C10	125.2(4)		124.4(3)	
C9–N2	1.374(5)	1.374(4)	C8–C9–N2	108.8(3)	359.9(6)	110.1(3)	359.9(5)
C9–C10	1.402(6)	1.404(4)	N2–C9–C10	125.9(4)		125.4(3)	
C10–C17	1.498(4)	1.494(4)	C9–C10–C1A[a)]	126.6(4)		125.7(3)	
			C9–C10–C17	116.4(3)	360.0(6)	118.0(2)	360.0(5)
			C1A–C10–C17	117.0(3)		116.3(3)	
